# *Cj1411c* Encodes for a Cytochrome P450 Involved in *Campylobacter jejuni* 81-176 Pathogenicity

**DOI:** 10.1371/journal.pone.0075534

**Published:** 2013-09-26

**Authors:** Luis A. J. Alvarez, Billy Bourke, Gratiela Pircalabioru, Atanas Y. Georgiev, Ulla G. Knaus, Simon Daff, Nicolae Corcionivoschi

**Affiliations:** 1 National Children’s Research Centre, Our Lady's Children's Hospital Crumlin, Dublin, Ireland; 2 Conway Institute, School of Medicine and Medical Science, University College Dublin, Dublin, Ireland; 3 School of Chemistry, University of Edinburgh, Edinburgh, Scotland, United Kingdom; 4 Banat’s University of Agricultural Sciences and Veterinary Medicine, School of Animal Sciences and Biotechnology, Timişoara, Romania; Institut National de la Recherche Agronomique, France

## Abstract

Cytochrome P450s are *b*-heme-containing enzymes that are able to introduce oxygen atoms into a wide variety of organic substrates. They are extremely widespread in nature having diverse functions at both biochemical and physiological level. The genome of *C. jejuni* 81-176 encodes a single cytochrome P450 (*Cj1411c*) that has no close homologues. *Cj1411c* is unusual in its genomic location within a cluster involved in the biosynthesis of outer surface structures. Here we show that *E. coli* expressed and affinity-purified *C. jejuni* cytochrome P450 is lipophilic, containing one equivalent Cys-ligated heme. Immunoblotting confirmed the association of cytochrome P450 with membrane fractions. A *Cj1411c* deletion mutant had significantly reduced ability to infect human cells and was less able to survive following exposure to human serum when compared to the wild type strain. Phenotypically following staining with Alcian blue, we show that a *Cj1411c* deletion mutant produces significantly less capsular polysaccharide. This study describes the first known membrane-bound bacterial cytochrome P450 and its involvement in *Campylobacter* virulence.

## Introduction

During the past few decades *Campylobacter* has been identified as the most common cause of bacterial diarrheal illness in the European Union and worldwide [1]. This pathogen is also responsible, in some patients, for severe neurological diseases including Guillain-Barré syndrome [2]. Over 95% of campylobacteriosis cases are endemic, the rest attributable to outbreaks, usually due to contaminated private water supplies or unpasteurised milk [3]. *Campylobacter jejuni* 81-176 was originally isolated from a 9-year-old girl with diarrhea, and it was shown to cause severe disease in human patients [4].

Cytochromes P450s are a superfamily of proteins with a maximum absorption at 450 nm and are characterized by the presence of a conserved Cys residue [5]. This cysteine distinguishes cytochrome P450s from other oxygen activating enzymes such as globins and peroxidases that utilize histidine during the reaction with hydroperoxide [6]. The number of cytochrome P450 proteins encoded in bacteria is variable. *Escherichia. coli*, for example, has no cytochrome P450 encoded in its genome, whereas *Mycobacterium tuberculosis* contains twenty and *Streptococcus* *coelicolor* eighteen P450 cytochromes [7]. . The bacterial cytochrome P450 enzymes have functions that include camphor degradation [8] and biotin synthesis [9]. The genome of *C. jejuni* 81-176 encodes a single cytochrome P450, *Cj1411c* (CYP1411c).

The CYP1411c coding sequence is 1359 bp long (453 amino acids). It is located in a genomic region that mainly encodes proteins known to be involved in bacterial outer surface biosynthesis, and amino-acid transferases. The function of the *Campylobacter* enzyme cannot be inferred from sequence comparisons, but the location of the coding sequence at the downstream end of the large *Kps* gene region (capsule biosynthesis cluster) indicates a possible function in the biosynthesis of cell surface components [10].

Outer surface structures are important in *Campylobacter* pathogenesis. They have been shown to be responsible for adherence and invasion, colonization and disease, maintenance of cell surface charge and serum resistance [11-14]. In this study we show that CYP1411c is a membrane-bound cytochrome P450, that when mutated significantly impairs *C. jejuni* 81-176 pathogenicity. In addition, CYP1411c protein expression was increased following cellular internalization and diminished capsular polysaccharides (CPS) on the outer surface of the CYP1411c deletion mutant was detected.

## Materials and Methods

### Bacterial strains and growth conditions

*C. jejuni* 81-176 wild type and the deletion mutant *Campylobacter jejuni* 81-176 Δ*Cj1411c* were grown on Mueller-Hinton (MH) Agar or in MH Broth (OXOID, UK) at 37°C for 48h under microaerobic conditions (5% CO_2_, 5% O_2_, 90% N_2_). HCT-8 cells (human adenocarcinoma cells) were maintained in RPMI 1640 (Sigma, UK) containing 10% fetal bovine serum. Cells were grown at 21% O_2_, 5% CO_2_ and transferred to microaerobic conditions for infection studies. The *E. coli* strains Nova Blue [*endA1 hsdR17* (r_K12_^–^ m_K12_^+^) *supE44 thi-1 recA1 gyrA96 relA1 lac* F[*proA* ^*+*^ *B* ^*+*^ *lacI* ^*q*^ *Z*Δ*M15*::Tn*10*] (Tet^R^)] or TUNER [F^-^ *ompT hsdS*_B_ (r_B_^–^ m_B_^–^) *gal dcm lacY1*(DE3) pLacI (Cam^R^)] were maintained in either LB Broth or agar with appropriate antibiotics.

### Mutant Construction

To examine the role of CYP1411c in *C. jejuni* virulence we have constructed a deletion mutant as previously described [15]. Two DNA fragments of 400 base pairs were each amplified by PCR from upstream (P1FOR450 and P1REV450) and downstream (P2FOR450 and P2REV450) of the *Cj1411c* gene. The chloramphenicol cassette originating from PYR112 was inserted by overlapping PCR (CMFOR and CMREV) between the two 400 base pair DNA fragments. Primer sequences are shown in Table 1. The resulting deletion cassette was used to transform *C. jejuni* 81-176 by natural transformation [16]. For reconstitution the *Cj1411c* gene was cloned into the SmaI site of PRY107 (Km^R^) plasmid (a non-suicidal plasmid) and transformed by natural transformation into the *C. jejuni* 81-178 Δ*Cj1411c* strain to give *C. jejuni* 81-178 Δ*Cj1411c*::*Cj1411c* strain.

**Table 1:** Primers used.

Primer name	Primer sequence 5’–3’
P1 FOR450	GACAAAATTTAGATTTATCTTTACTTCATAAGTTAAAAATTC
P1 REV450	CTTGGAAAGGAACACCGCCGAGATTTTTACCTTTTCTTCTAA
P2 FOR450	ACCCTTTAGGAACTAAAGGGCGAAAGAGTTAGAACTAAAA
P2 REV450	TGCTTGTCTAAATTTCTTGAAAGTATGAAATCCACCACAGGT
CM FOR	CTCGGCGGTGTTCCTTTCCAAG
CM REV	CGCCCTTTAGTTCCTAAAGGGT
P450 FOR	ATGAGTGAATGCCCCTTTTTTCCAAAACCTTATAAAAATAAAGC
P450 REV	TCAGTGGTGGTGGTGGTGGTGTAGCTTTCTTTTGCTAAATTTTAT

### Protein expression, purification and antibody production

For antibody production the *Cj1411c* gene was amplified from *C. jejuni* 81-176 genomic DNA using primers P450 FOR and P450 REV. Following amplification by PCR the recombinant DNA fragment was ligated into the EcoRV site of the pETBlue1 vector. For initial cloning the ligated vector was transformed in *E. coli* Nova Blue and for overexpression in *E. coli* TUNER. The overexpression strain was grown in Terrific Broth with carbenicillin/chloramphenicol at 37 °C until OD_600_ = 0.5 and induced with 1mM IPTG overnight at room temperature. Bacterial pellets were harvested and lysed in 25 mM Tris pH7.5, 250 mM NaCl, 25 mM Imidazole, 1 mM DTT, 0.1% Triton X-100 and protease inhibitors (Sigma, UK). *E. coli* lysates were loaded onto a HisTrapTM FF column and eluted with 25 mM Tris 7.5, 250mM NaCl, 0.1% Triton, 1mM DTT on an AKTApurifier®. Collected samples were desalted and concentrated on Centricon® devices with a MW 10.000 cut-off and stored in 25 mM Tris-HCL pH 7.5 at -80°C. Protein identity, purity and phosphorylation state was analyzed by Coomassie blue. The protein was eluted with a gradient of 25 mM Tris pH 7.5, 250 mM NaCl, 250 mM Imidazole, 1 mM DTT, 0.1% Triton X100 and concentrated to 5-10 ml. In order to obtain a higher purity the concentrated sample was additionally purified on a Superdex S200 FPLC column in 25 mM Tris pH7.5, 250 mM NaCl, 1 mM DTT, 0.1% Triton X100. The eluted protein was concentrated to a volume of approximately 5 ml (100µM). The purified protein was used for antibody production at Capra Science (Sweden).

### Pathogenicity assay

The gentamicin protection assay was used to test the role of P450 cytochrome in the ability of *C. jejuni* 81-176 to adhere and invade host epithelial cells [14]. Briefly, HCT-8 cells were grown (60% confluence) for 15 to 18 h in six-well tissue culture plates at a concentration of 1 × 10^5^ cells per well. Plate grown *C. jejuni* 81-176 wild type and *C. jejuni* 81-178 Δ*Cj1411c* mutant were washed and re-suspended in tissue culture medium at an OD_600_ of 0.4. The HCT-8 cells were washed with PBS, and 2 ml of fresh culture medium was added to each well. Bacteria were added to give a multiplicity of infection of 10. Tissue culture plates were centrifuged at 250 × g for 5 min and incubated for 3 h at 37°C in 10% CO_2_. To quantify the number of cell-associated bacteria, infected monolayers were washed at least three times with PBS and treated with 0.1% Triton X-100 in PBS at 37°C for 30 minutes. Tenfold dilutions of each well were plated onto the appropriate agar and colonies enumerated after 3 days of incubation. To quantify the number of bacteria that invaded HCT-8 cells, the infected monolayers were washed with PBS and tissue culture medium (2 ml) containing gentamicin (400 μg/ml) was added to half the wells, and medium with no antibiotic was added to the remaining wells. The tissue culture plates were then incubated for a further 2 h at 37°C and washed with PBS. HCT-8 cells were lysed by the addition of 100μl of 0.1% Triton X-100 in PBS and incubated for 10 to 15 min at 37°C. Tenfold dilution of the contents of each well was plated onto the appropriate agar and colonies were enumerated after 3 days of incubation. Invasion efficiency was calculated as the average of the total number of CFU/total initial inoculum. *C. jejuni* 81-176 passaged in RPMI 1640 (without cells) was also tested for the ability to adhere to and invade HCT-8 cells. All assays were conducted in triplicate and repeated independently three times. The significance of differences in adhesion and invasion between samples was determined using the Student t test. A P value of <0.05 was defined as significant.

### Cell infection for RNA extraction

HCT-8 cells were seeded on 12 well plates at a density of 10^8^/well and allowed to grow overnight to 60-70% confluence. Agar-grown bacteria were used to prepare the inocula for infection experiments. Bacteria were harvested from plates and suspended in RPMI 1640 medium. Six well plates, containing HCT-8 cells, were inoculated with 1 ml of bacterial suspension with OD_600_ of 0.4. HCT-8 cells were infected at 37°C under microaerobic conditions (5% O_2_, 5% CO_2_, and 90% N_2_) for 1.5 and 3h. Viable counts were determined for inocula at each passage (both with and without host cells) to ensure that similar numbers of live bacteria were present at each stage of infection and for non-infected controls.

### RNA extraction and qPCR

Total RNA was isolated from internalized bacteria by using the RNeasy Kit (Qiagen). The RNA was reverse transcribed using the High Capacity cDNA reverse transcription kit (Applied Biosystems, CA) according to the manufacturer’s protocol. The mRNA levels were determined by quantitative RT-PCR using SYBR Green (Applied Biosystems, CA) on a 7900 Fast Real-Time System (Applied Biosystems, CA) and the primers qP450F (5’-atgagtgaatgccccttttttcca-3’) and qP450R (5’-ctcccctaaaagcggacttaaaag-3’). Relative quantity of p450 mRNA was calculated using the ΔCt method. Universal eubacterial primers for rRNA 16S were used as an endogenous control (UniF340-5’-actcctacgggaggcagcagt-3’ and UniR514 -5’attaccgcggctgctggc-3’). For each experiment the RNA was extracted from 6 wells of infected cells and also from the bacteria in control experiments in the absence of cells.

### CPS staining

CPS was prepared from bacteria as described by Hitchcock [17]. Bacteria were harvested by centrifugation and suspended in 100 μl of lysis buffer containing 31.25 mM Tris-HCl (pH 6.8), 4% sodium dodecyl sulfate, 0.025% bromophenol blue, and 20% glycerol. After heating to 100°C for 5 min, 20 μl of the sample was transferred into a fresh tube, 1 μl of 20 mg/ml proteinase K was added to the solution, and the tubes were incubated for 1 h at 50°C. The samples were fractionated on NuPage Novex 10% bis-Tris gels (Invitrogen, United Kingdom). Following electrophoresis, gels were stained with Alcian blue (0.1% Alcian blue in 40% ethanol, 5% acetic acid) [18].

### Serum resistance assay

The sensitivity of bacteria to human serum was measured [19]. *C. jejuni* 81-176 wild type and *Cj1411c* deletion mutant were plate-grown and resuspended in fresh tissue culture medium (RPMI 1640) to give an OD_600_ of 0.1. Five microliters of bacterial suspension was added to duplicate wells of a six-well plate containing 800 μl of Mueller-Hinton broth and 200 μl of active pooled human serum or to separate wells containing 800 μl of Mueller-Hinton broth and 200 μl heat-inactivated human serum to give a total volume of 1ml. The plates were incubated for 1 h at 37°C under microaerobic conditions. Bacteria from each well were diluted 10-fold and 50µl plated on dry Mueller-Hinton agar plates. The plates were incubated at 37°C under microaerobic conditions. Colony counts were performed after an incubation time of 2 days. All assays were conducted in triplicate and repeated independently three times.

### Western Blotting

Proteins were separated on 10% SDS polyacrylamide gels and transferred to 0.45 µm nitrocellulose membrane (Millipore). Following WB the membranes were blocked in 3% BSA + 0.05% Tween for 30 minutes. Primary antibodies against *Cj1411c* P450 (rabbit polyclonal), anti-His (sc-53073, Santa Cruz Biotechnology) were used. The anti-CadF and anti-Fur antibodies (rabbit polyclonal) confirmed the purity of our membrane fractionations. Following washing in PBS-TWEEN the membranes were incubated with the secondary antibodies (goat anti-rabbit - #7074S, Cell Signaling; goat anti-mouse sc-2031, Santa Cruz). Detection was performed with the PIERCE chemiluminiscence kit (Thermo, Fisher Scientific). Densitometry was performed using Fiji ImageJ software.

### Subcellular fractionation

Bacterial cellular fractionation was performed as previously described [20]. Bacteria were harvested by centrifugation in 20mM Tris (pH 7.5) at 12000g for 20 minutes at 4°C followed by washing 3 times in 20mM Tris (pH 7.5). The bacteria were then disrupted by sonication in 20mM Tris (pH 7.5) with protease inhibitors (Sigma, UK). In order to collect unbroken cells we have centrifuged the sonicated material for 10 minutes at 2000 rpm and kept the supernatant. In order to collect the total membranes the sonicated material was centrifuged at 40000g for 1 hour at 4°C and the supernatant containing the cytosolic fraction was collected. In order to separate the inner membranes from the outer membranes the resulting pellet was resuspended in 20mM Tris (pH 7.5) containing 2% sodium lauryl sarcosine and incubated for 30 minutes at 4°C. Following centrifugation at 40000g for 1 hour at 4°C the supernatant fraction contains the inner membranes and the outer membranes are in the pellet. The outer membranes were re-suspended in 20mM Tris (pH 7.5). The purity of our membrane fractionations was controlled using an anti-Fur antibody (a cytosolic protein) and anti-CadF antibody (an outer membrane protein).

### UV-visible spectroscopy

The visible absorption spectra for the purified cytochrome P450 protein were recorded by using a Shimadzu UV-1650 dual-wavelength/double beam spectrophotometer. The measurement was performed in a 1 cm path length quartz cuvette containing 1µM purified protein in 25mM Tris (pH 7.5) and 200mM KCl. The spectra was recorded between 800-300 nm. For the carbon monoxide (CO) bound spectra CO was bubbled through the sample for one minute and the spectrum was recorded as mentioned above.

## Results

### 1. Spectroscopic characterization of the purified protein

**Figure 1:**
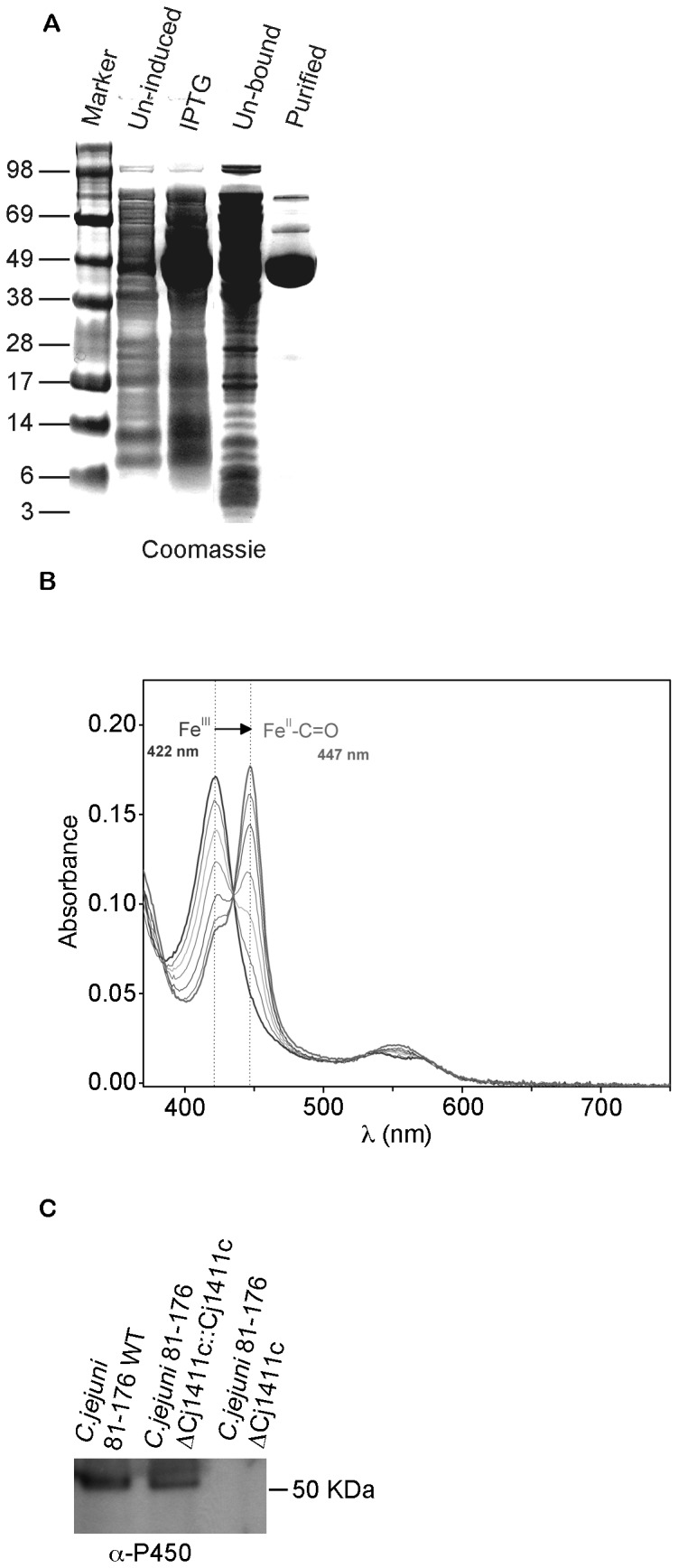
*Cj1411c* protein and mutant characterization. (a) Visualization of protein expression using 10% SDS-PAGE and stained with Coomassie Blue (b) Identification of the 450 nm absorption in the SORET band with CO bound on recombinant CYP1411c. Successive spectra show the oxidation transition from a 420 nm peak to a 450 nm peak in CYP1411c following exposure to CO. (c) Western blot analysis with an anti-CYP1411c antibody following gene deletion showing the absence of the cytochrome P450 protein in *C. jejuni 81-176* Δ*Cj1411c*.

To analyze the cellular localization of *Cj1411c* and its biochemical characteristics purified active enzyme and anti-CYP1411c antibody were essential. The CYP1411c protein was overexpressed in *E. coli* Tuner DE3 and purified by 2-step chromatography. Figure 1(a) shows the successfully purified cytochrome P450 protein following Coomassie blue staining of SDS gels. Following successful overexpression and purification we raised an antibody against CYP1411c for further characterization by Western blotting, (Figure 1c). Spectroscopy showed a heme Soret band at 422 nm and a spectrum characteristic of a low spin ferric active cytochrome P450 (Figure 1b). Addition of 1 mg sodium dithionite caused no change to the spectrum, indicating that reduction of the heme to the ferrous form is unfavourable under these conditions [6]. The fact that the heme is unable to be reduced in the purified enzyme suggests that substrate binding will act as a trigger for reduction, as is common in cytochrome P450s, by inducing a reduction potential shift [6]. This can only be tested once the substrate has been identified. Reduction potential can also be influenced by environment, and it is possible that the enzyme needs to be associated with a membrane to be functional in this respect.

### 2. The role of *Cj1411c* in *Campylobacter jejuni* 81-176 pathogenicity

In order to investigate the role of CYP1411c in *C. jejuni* 81-176 virulence we constructed a deletion mutant (*C. jejuni* 81-176 ∆*Cj1411c*). The deletion strain was reconstituted using the PYR107 shuttle plasmid (*C. jejuni* 81-176 ∆*Cj1411c*::*Cj1411c*) The successful *Cj1411c* gene deletion was confirmed by Western blotting using an anti-P450 antibody (Figure 1c).

**Figure 2:**
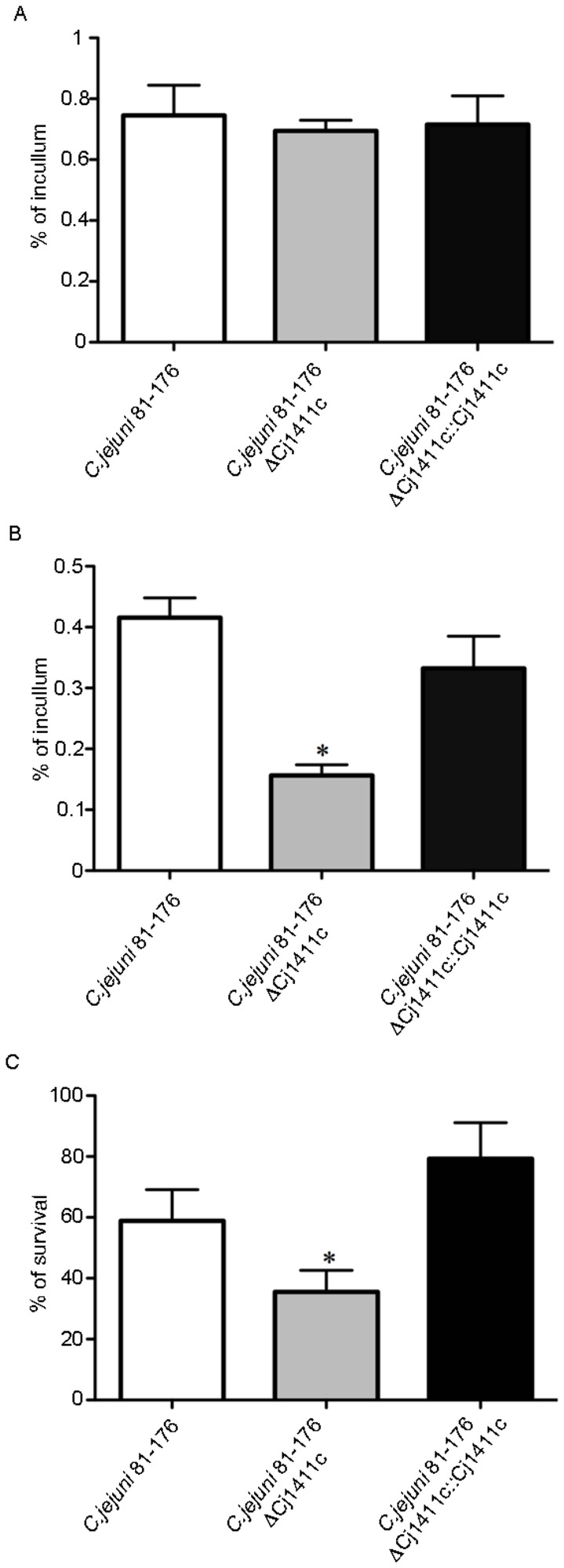
Virulence and serum resitance. (a) adhesion to HCT-8 cells, (b) invasion of HCT-8 cells of *C. jejuni* 81-176 wild type, *C. jejuni* 81-176 Δ*Cj1411c* and *C. jejuni* 81-176 Δ*Cj1411c*::*Cj1411c*. (c) serum resistance - the survival rate is defined as the number of *C. jejuni* 81-176 colonies isolated following exposure to human serum divided by the number of colonies surviving in heat inactivated serum, expressed as a percentage. Statistical significance (Student’s *t* test) relative to the level of wild type strain is indicated. *P=0.001. The experiments were done in triplicate and on three separate occasions. The error bars represent standard deviations for six separate wells.

Because cytochrome P450s are associated with pathogenicity in other organisms we investigated the role CYP1411c in virulence by comparing the adhesion (Figure 2a), invasion (Figure 2b) of the wild type strain with the CYP1411c deletion mutant and also with the reconstituted mutant. Following gentamicin protection assay, the deletion mutant showed a significant reduction in invasion (P = 0.005), with the virulence being restored following reconstitution. Levels of adhesion were unaffected.

We also tested the effect of CYP1411c gene deletion on serum resistance as it is directly connected to virulence. The wild type strain had an average rate of survival of 60%. However, there was a dramatic decrease (23%) in the rate of survival of the cytochrome P450 deletion strain (P < 0.003). The reconstituted strain showed restored survival when compared to the wild type strain (Figure 2c). These results demonstrate that the CYP1411c is involved in *C. jejuni* 81-176 pathogenicity by contributing to its virulence and serum resistance.

### 3. CYP1411c gene expression is up-regulated following invasion of epithelial cells

**Figure 3:**
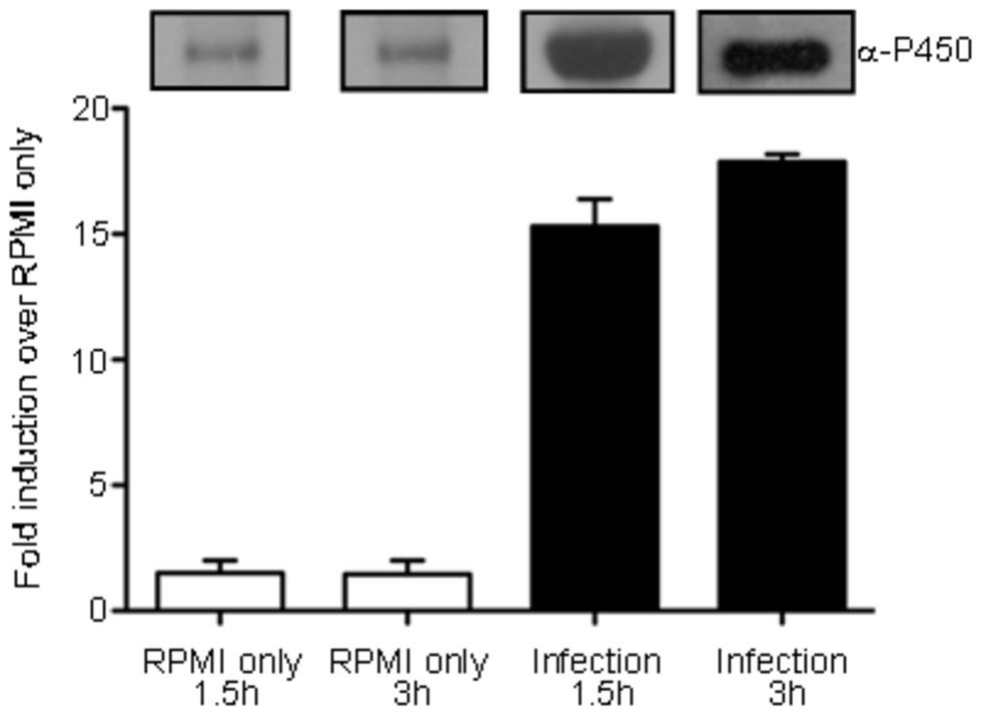
qRT-PCR analysis of *Cj1411c* gene expression during infection. *Cj1411c* expression analysis in *C. jejuni* 81-176 following infection of HCT-8 cells. The gene expression level for 1.5h in RPMI was set to 1 as the basal level. All other levels are expressed as fold change over the basal gene expression. Error bars represent ±S.D. of 3 independent experiments. The corresponding protein expression levels assessed by Western blot with the anti-P450 antibody are shown.

Next we have investigated the hypothesis that *Cj1411c* is important during survival inside the host following internalization. In order to test this hypothesis we isolated RNA from bacteria that had infected human epithelial HCT-8 cells for intervals of 1.5 and 3 hours. We show, using q-PCR, that the CYP1411c gene expression levels were significantly increased when *C. jejuni* attached to or internalized in human intestinal epithelial cells. In the absence of HCT-8 cells the gene expression levels remained low whereas the 16S rRNA levels remained constant (data not shown) (Figure 3). Investigation of protein levels by Western blotting using the anti-CYP1411c antibody confirmed the q-PCR results (Figure 3). These results show that CYP1411c is up-regulated after internalization, suggesting that this enzyme is metabolically required when the pathogen invades the host. This could explain the significant decrease in invasiveness of the deletion mutant (Figure 2b).

### 4. CYP1411c a membrane-associated cytochrome P450

**Figure 4:**
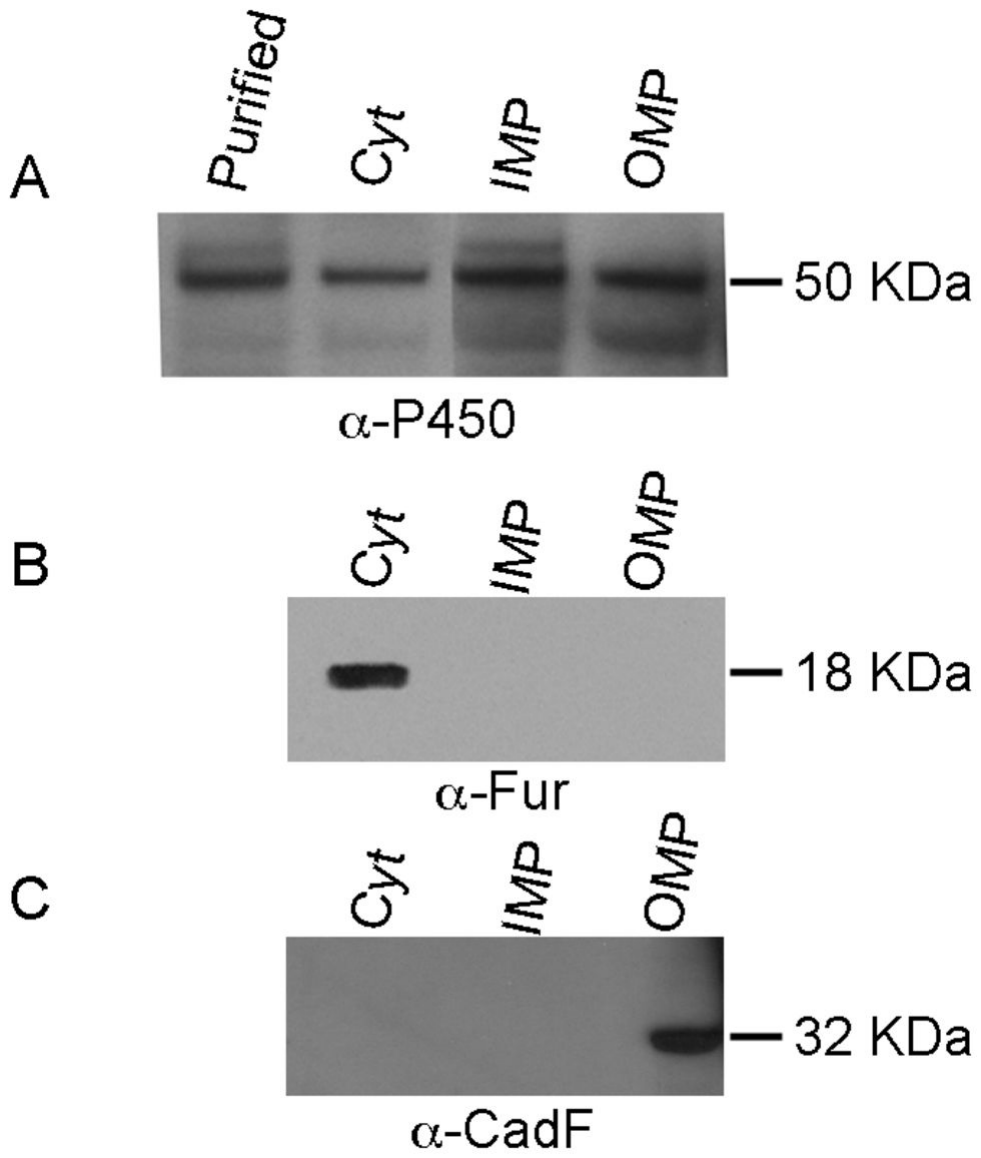
CYP1411c cellular localization. (A) Anti-P450 immunoblotting on bacterial fractions (cytosolic fraction, Cyt; inner membrane protein, IMP and outer membrane protein fraction, OMP). Recombinant CYP1411c protein (purified) was used as a positive control. Fractions were validated with an antibody recognizing the cytosolic Fur protein (B) and the outer membrane protein CadF (C).

**Figure 5:**
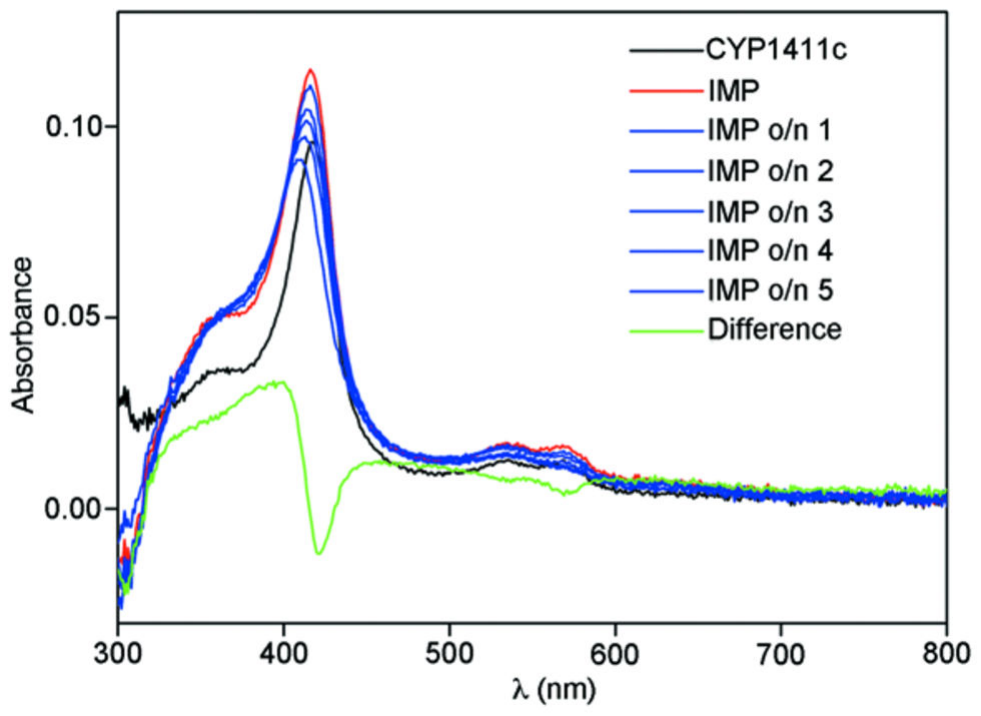
CYP1411c spectral change following incubation with the inner membrane fraction. IMP o/n 1-5 represent successive spectral recordings following incubation of purified CYP1411c protein with *C. jejuni* 81-176 inner membrane proteins. Spectra were recorded at 1 min intervals.

In order to investigate the subcellular localization of CYP1411c we have separated cytosolic, inner membrane and outer membrane proteins from *C. jejuni* 81-176. Western blotting on the individual fractions was performed using the anti-P450 antibody (Figure 4A). CYP1411c was present in all three fractions, including the inner and outer membrane. Densitometry on blots showed that 83% of the total CYP1411c present in *C. jejuni* 81-176 was membrane bound. As the majority of CYP1411c is membrane bound, its redox partners or possible substrates should be localized within the same subcellular fraction. In order to check the purity of bacterial fractionations we have used an anti-Fur antibody, as Fur protein is a known cytosolic protein (Figure 4B) and an anti-CadF antibody detection of CadF, a known outer membrane protein (Figure 4C). Following bacterial fractionation we investigated by spectroscopy the effect of membrane proteins on purified CYP1411c absorption spectra. The purified enzyme was incubated with *C. jejuni* 81-176 inner membrane fractions for overnight, followed by the addition of dithionite, which caused a slow shift in the Soret band from 422 to 400 nm and the merging of the α and β bands. Such an effect is characteristic of a low to high spin transition in cytochrome P450s, often observed on substrate binding (Figure 5). The spin-state change facilitates reduction of the protein, which was also observed in this case. Our results show that *C. jejuni* 81-176 inner membrane proteins can cause an alteration in spectra, suggesting an interaction with purified CYP1411c. Such changes in spectra were not observed when probed with cytosolic fractions from *C. jejuni* 81-176. This result confirms that either the redox partner or the substrate of CYP1411c is localized within this bacterial fraction and is important for enzyme function. This data identifies the CYP1411c as the first bacterial cytochrome P450 to be membrane-associated.

### 5. CYP1411c is involved in outer surface biosynthesis

**Figure 6:**
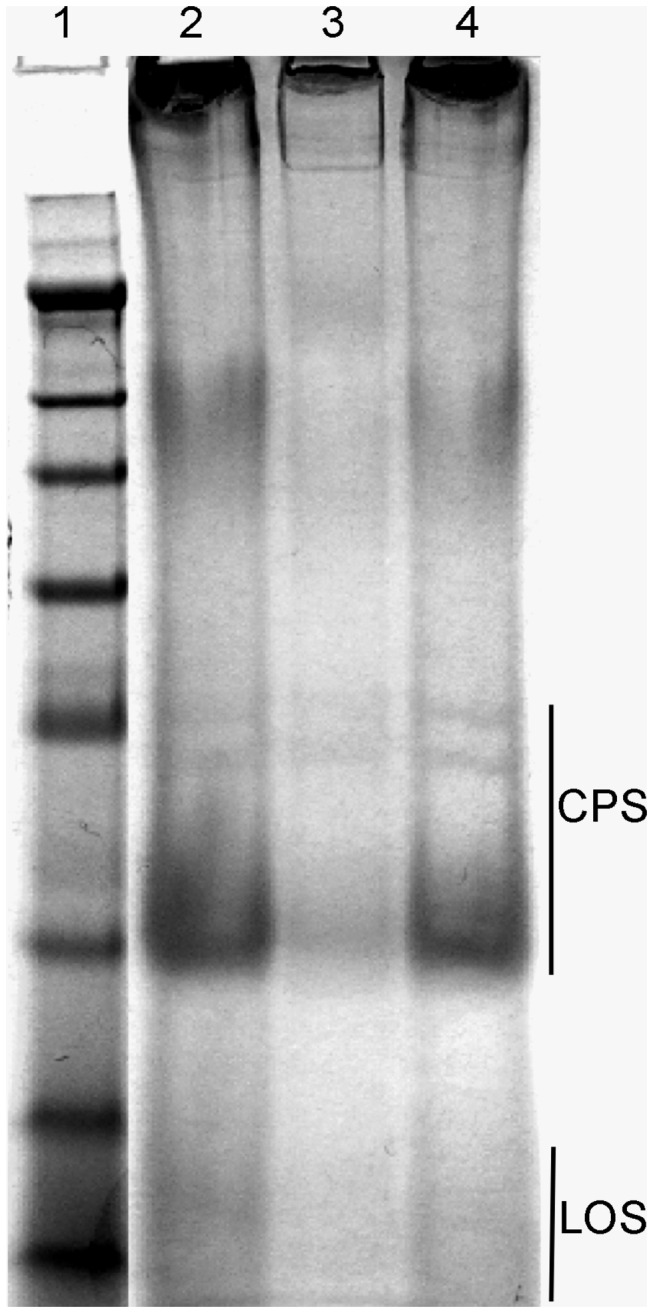
The effect of *Cj1411c* gene deletion on CPS production. Alcian blue stained CPS of *C. jejuni* 81-176 wild type (lane 2) was compared with the CPS extracted *C. jejuni* 81-176 Δ*Cj1411c* (lane 3) and *C. jejuni* 81-176 Δ*Cj1411c*::*Cj1411c* (lane 4).

In light of the unique position of this cytochrome P450 in the capsule biogenesis locus, and the effect of deleting *Cj1411c* on pathogenicity, we hypothesized that the cytochrome P450 protein is involved in or contributes to CPS (capsule polyssacharide) production. We extracted CPS from *C. jejuni* 81-176 wild type, Δ*Cj1411c* deletion mutant and Δ*Cj1411c*::*Cj1411c* reconstituted mutant. Following separation in SDS gels, staining with Alcian blue was performed. As depicted in Figure 6 the CYP1411c mutant (Figure 6, lane 3) displayed significantly reduced CPS and LOS staining when compared to the wild type strain (Figure 6, lane 2). The recovery of staining in the reconstituted mutant (Figure 6, lane 4) indicates clearly that CYP1411c impacts on the biosynthesis of these structures.

## Discussion

Cytochrome P450s comprise an important group of proteins involved in the synthesis of physiologically active compounds, drug metabolism and bioconversion of xenobiotics. Although the *C. jejuni* 81-176 genome database annotation identifies only one cytochrome P450, no data are available on the possible functions of this protein. Overexpression of the protein in *E. coli* and antibody production allowed us to identify this cytochrome not only in cytosolic fractions but also in membrane fractions. This is consistent with data obtained from bioinformatics that place this cytochrome within a genomic cluster involved in biosynthesis of outer surface structures (http://www.sanger.ac.uk). While other known prokaryotic cytochrome P450s are cytosolic proteins, the eukaryotic enzymes are known to be integral membrane proteins [21]. This common feature of mammalian cytochrome P450s is important, because their catalytic domain is facing the cytosol allowing them to bind liposoluble substrates [22]. In our experiments we have identified Cj1411c as being predominantly localized in the outer membrane fraction. While not unambiguously confirming this location, the control experiments, using known cytosolic and membrane proteins, support this finding. We speculate that the cytochrome P450 enzyme substrate is membrane localized in *Campylobacter* and that the cytosolic fraction of the CYP1411c enzyme might only be required during the catalytic cycle in electron scavenging. These hypotheses require further investigation.

The biochemical studies performed on purified protein suggest that the reduction potential of the heme is very low. A similar situation is observed with the F393W mutant of P450BM3 [6]. A slow shift in the Soret band to 447 nm indicated formation of the cytochrome P450 ferrous CO complex characteristic of Cys-thiolate ligated heme when CO gas was added to the sample (Figure 1B). Thus, CO is able to induce the heme into the ferrous form, not accessible in the presence of dithionite only. Observation of a Cys-ligated ferrous heme CO complex confirms that CYP1411c is a typical cytochrome P450, and was purified with the protein-heme ligand complex intact. The change in absorption following interaction with inner membrane fractions represents a key result for its functionality. It is important for cytochrome P450s to have redox partners in the vicinity because they require two electrons for their catalytic function and these are usually supplied in two consecutive steps from NAD(P) H via the redox partners. Interestingly one of the predicted redox partners for CYP1411c is an iron-sulfur protein (*Cj0074c*), known to be localized within the inner membrane fraction [23].

In *C. jejuni* 81-176, outer surface components are actively involved in mediating its pathogenicity. The unique localization of CYP1411c at the end of the surface biosynthesis cluster suggested its possible association with bacterial membranes and involvement in the formation of surface components. The CYP1411c deletion mutant is significantly less virulent than wild type *C. jejuni* 81-176. The decrease in virulence was accompanied by loss of resistance to human serum. One of the outer surface features associated with attenuated virulence is the capsule polysaccharide (CPS). The CYP1411c deletion mutant produced significantly less CPS than the wild type *C. jejuni* 81-176 strain. Previous studies demonstrated that loss of CPS can impair the ability of *C. jejuni* to invade human cells providing a possible explanation for the attenuation of *C. jejuni* ∆*Cj1411c* invasiveness [14,20,24,25].

The significant increase in gene and protein expression of CYP1411c after invasion of the host, associated with the loss in pathogenicity of the deletion mutant, highlights the importance of this cytochrome P450 in host-pathogen interaction. Involvement of CYP1411c in pathogenicity has been described also in other pathogens. For example *M. tuberculosis* CYP125 is involved in catabolism of host cholesterol generating immunomodulatory compounds to increase survival in the host [26]. The role of cytochrome P450s in survival within the host goes beyond the bacterial regnum. A similar role was shown for CYP5122A1, a cytochrome P450 from the parasite *Leishmania donovani*. In this parasite CYP5122A1 was linked to processes including cell growth, infection and ergosterol biosynthesis [27].

## Conclusion

In summary *C. jejuni* CYP1411c is uniquely located in the membrane fractions of this organism and plays a role in CPS production. Its role in pathogenicity points to its potential as a drug target. Further studies are required to elucidate the complex biological role of this protein.
